# Evidence of novel miR-34a-based therapeutic approaches for multiple myeloma treatment

**DOI:** 10.1038/s41598-017-18186-0

**Published:** 2017-12-20

**Authors:** Mayra Rachele Zarone, Gabriella Misso, Anna Grimaldi, Silvia Zappavigna, Margherita Russo, Evzen Amler, Maria Teresa Di Martino, Nicola Amodio, Pierosandro Tagliaferri, Pierfrancesco Tassone, Michele Caraglia

**Affiliations:** 1 Department of Biochemistry, Biophysics and General Pathology, University of Campania “L. Vanvitelli”, Naples, Italy; 2 Institute of Biophysics, 2nd Faculty of Medicine, Charles University, Prague, Czech Republic; 3 Laboratory of Tissue Engineering, Institute of Experimental Medicine, Czech Academy of Sciences, Prague, Czech Republic; 4 Department of Experimental and Clinical Medicine, Magna Graecia University, Salvatore Venuta University Campus, Catanzaro, Italy; 5 Sbarro Institute for Cancer Research and Molecular Medicine, Center for Biotechnology, College of Science and Technology, Temple University, Philadelphia, Pennsylvania USA

## Abstract

MiR-34a acts as tumor suppressor microRNA (miRNA) in several cancers, including multiple myeloma (MM), by controlling the expression of target proteins involved in cell cycle, differentiation and apoptosis. Here, we have investigated the combination between miR-34a and γ-secretase inhibitor (γSI), Sirtinol or zoledronic acid (ZOL) in order to enhance the inhibitory action of this miRNA on its canonical targets such as Notch1 and SIRT1, and on Ras/MAPK-dependent pathways. Our data demonstrate that miR-34a synthetic mimics significantly enhance the anti-tumor activity of all the above-mentioned anti-cancer agents in RPMI 8226 MM cells. We found that γSI enhanced miR-34a-dependent anti-tumor effects by activating the extrinsic apoptotic pathway which could overcome the cytoprotective autophagic mechanism. Moreover, the combination between miR-34a and γSI increased the cell surface calreticulin (CRT) expression, that is well known for triggering anti-tumor immunological response. The combination between miR-34a and Sirtinol induced the activation of an intrinsic apoptotic pathway along with increased surface expression of CRT. Regarding ZOL, we found a powerful growth inhibition after enforced miR-34a expression, which was not likely attributable to neither apoptosis nor autophagy modulation. Based on our data, the combination of miR-34a with other anti-cancer agents appears a promising anti-MM strategy deserving further investigation.

## Introduction

Multiple myeloma (MM) is a monoclonal tumor of bone marrow (BM) plasma cells (PCs) terminally differentiated. Monoclonal gammopathy of undetermined significance (MGUS), indolent multiple myeloma (IMM) and/or smoldering MM (SMM) are common premalignant tumors that precede MM. The evolution of these premalignant conditions into MM is dictated by multiple genetic and epigenetic events^1^ and the BM microenvironment could have a crucial role in fostering malignant transformation^2^. Several studies have shown that the BM microenvironment (BMM) promotes MM cell growth, survival and drug resistance through bidirectional interactions between MM cells and BM stromal cells or extracellular matrix^3^. Although the improvements of long-term outcome in MM treatment are observed, intrinsic or acquired drug resistance requires the development of new therapeutic strategies. The study of molecules regulating the cross-talk between MM cells and the BMM provides the basis to identify new possible target in order to inhibit MM development. Many evidences have been provided regarding MM microRNA (miRNA) signature, which includes miRNAs that could be associated with myeloma pathogenesis, suggesting a therapeutic potential in antagonizing the growth of transformed PCs^4–6^. MiRNAs are an evolutionarily conserved large class of noncoding RNAs, typically 18–22 nucleotides in length, acting as post-transcriptional repressors of target genes by antisense binding to their 3′ untranslated regions^7^. Several studies have reported that modulation of miRNA levels in MM cells impairs their functional interaction with the bone marrow microenvironment and produces a significant antitumor activity even able to overcome the protective bone marrow milieu^8^. In this regard, enforced expression of tumor suppressor microRNAs, such as miR-29b^9^, miR-23b^10^, miR-125b^11^, or inhibition of oncogenic miRNAs^12–14^ have demonstrated to trigger anti-tumor activity in preclinical models of MM.

In recent years, compelling evidence has demonstrated that miR-34a acts as a tumor suppressor in multiple types of cancers by controlling the expression of several target proteins involved in cell cycle, differentiation and apoptosis^15^. MRX34, a liposome-based miR-34a mimic is the first miRNA mimic to enter in clinic development and already evaluated in phase 1 clinical trial in cancer patients^16^. We recently demonstrated that enforced expression of miR-34a in MM cells induces modulation of several pathways, as ERK and Akt-dependent signaling, which have specific relevance in MM pathobiology^12^. In addition, our group has demonstrated the anti-MM effects induced by miR-34a, both *in vitro* and *in vivo*, using a nanotechnology-based delivery system. MiR-34a can be delivered in the tumor xenografts by direct intratumor or intravenous injection without producing toxicity in mice^12,17^. We reasoned that combination of miR-34a mimics with other anti-cancer agents could represent a novel way to develop new strategies based on miR-34a. On this basis, we have here investigated the combination between miR-34a and γ-secretase inhibitor (γSI), Sirtinol or zoledronic acid (ZOL) in order to enhance the inhibitory activity of this miRNA on its canonical targets such as Notch1 and SIRT1, and on Ras/MAPK-dependent pathways. Notch signaling is a highly conserved pathway that regulates cell-fate determination, stem cell self-renewal, proliferation and apoptosis^18^. In several malignancies, like T-cell acute lymphoblastic leukemia^19^, breast cancer^20,21^, melanoma^22^ and others, its deregulation has oncogenic effects. As a result, Notch signaling inhibitors offer a viable prospect for the treatment of several malignancies. One of the emerging approaches for blocking Notch signaling is to prevent the γ-secretase-mediated proteolytic cleavage that leads to the generation of Notch-IC intracellular domain. A number of γSIs have been assessed for their antitumor effects^23^ and a phase I clinical trial for a Notch inhibitor, MK0752 (developed by Merck, Whitehouse Station, NJ), has been launched for relapsed or refractory T-cell acute lymphocytic leukemia patients and advanced breast cancers^24^. A dual inhibition of Notch signaling through miR-34a and γSIs could therefore be a new targeted therapeutic strategy.

It was found that miR-34a inhibits the SIRT1, a NAD-dependent protein deacetylase Sirtuin-1 that regulates cellular senescence and limits longevity^25^. Activated SIRT1 deacetylates histones, histone methyl-transferases and a variety of non-histone targeted proteins, such as p53 and the retinoblastoma protein (Rb)^26^. The decrease in SIRT1 expression allows an increase in p53 acetylation and p53 activity. Several studies showed that p53 acts as a transcription factor to increase expression of a set of miRNAs that includes miR-34^27,28^. Yamakuchi and Lowenstein propose a positive feedback loop, in which p53 induces expression of miR-34a that suppresses SIRT1, increasing p53 activity^25^. Since increasing evidence shows that Sirtuins play a role in many biological processes, the development of small molecules that can regulate Sirtuins represents a promising anticancer strategy. Sirtinol, a β-naphthol-containing inhibitor, is reported to have anti-cancer activity in breast, lung, prostate and oral cancer cells^29^. We investigated anti-cancer effects on MM cells induced by Sirtinol adding robustness to p53-miR-34a-SIRT feedback loop.

Finally, we sought to investigate the effects induced by the ZOL in MM cells after transfection with miR-34a mimics. The medical management of MM bone disease is currently based on the clinical use of bisphosphonates (BP). Based on the significant reduction in the incidence of skeletal related events (hypercalcemia, compression fractures, pain and the necessity of radiotherapy/surgical intervention), ZOL was approved on February 2002, by the United States Food and Drug Administration for the treatment of patients with multiple myeloma in conjunction with standard antineoplastic therapy^30^. Moreover, several studies support the immediate treatment with ZOL also for potential anti-myeloma benefits; in fact, this BP acts, at least in part, by suppressing prenylation of small GTPases, including Ras. RAS/MAPK pathway plays a central role in MM disease, so its inhibition may be an efficient therapeutic strategy^31^ above all in conjunction with miRNAs (i.e.: miR-34a) interfering with other pathways representing an escape mechanism to RAS/MAPK-dependent signaling.

The main purpose of this study was to provide an innovative approach to MM treatment based on the inhibition of multiple components of the same signalling pathway, focusing on the potentiation of cell survival rate decrease induced by γSI, Sirtinol and ZOL through the ectopic expression of miR-34a in MM cells. We also investigated the molecular mechanisms underlying cell growth inhibition. In addition, being malignancies mostly a result from the disruption of the delicate balance between cell proliferation and elimination, the central goal of our study was to perform functional studies aimed to assess the death processes activated and their interplay in the combination setting. Knowledge about the crosslink between cell cycle progression and death processes, such as apoptosis and/or autophagy, can impact on the course of cancer treatment for the suppression of transformation and tumorigenesis. On the other hand, the recognition of the contribution of autophagy to tumorigenesis is also important since it is rarely obvious, given its paradoxical nature and its multiple effects on apoptosis.

## Results

### Cell survival rate decreases upon miR-34a transfection in combination with γ-SI or Sirtinol or ZOL treatment in RPMI 8226 cell line

**Figure 1:**
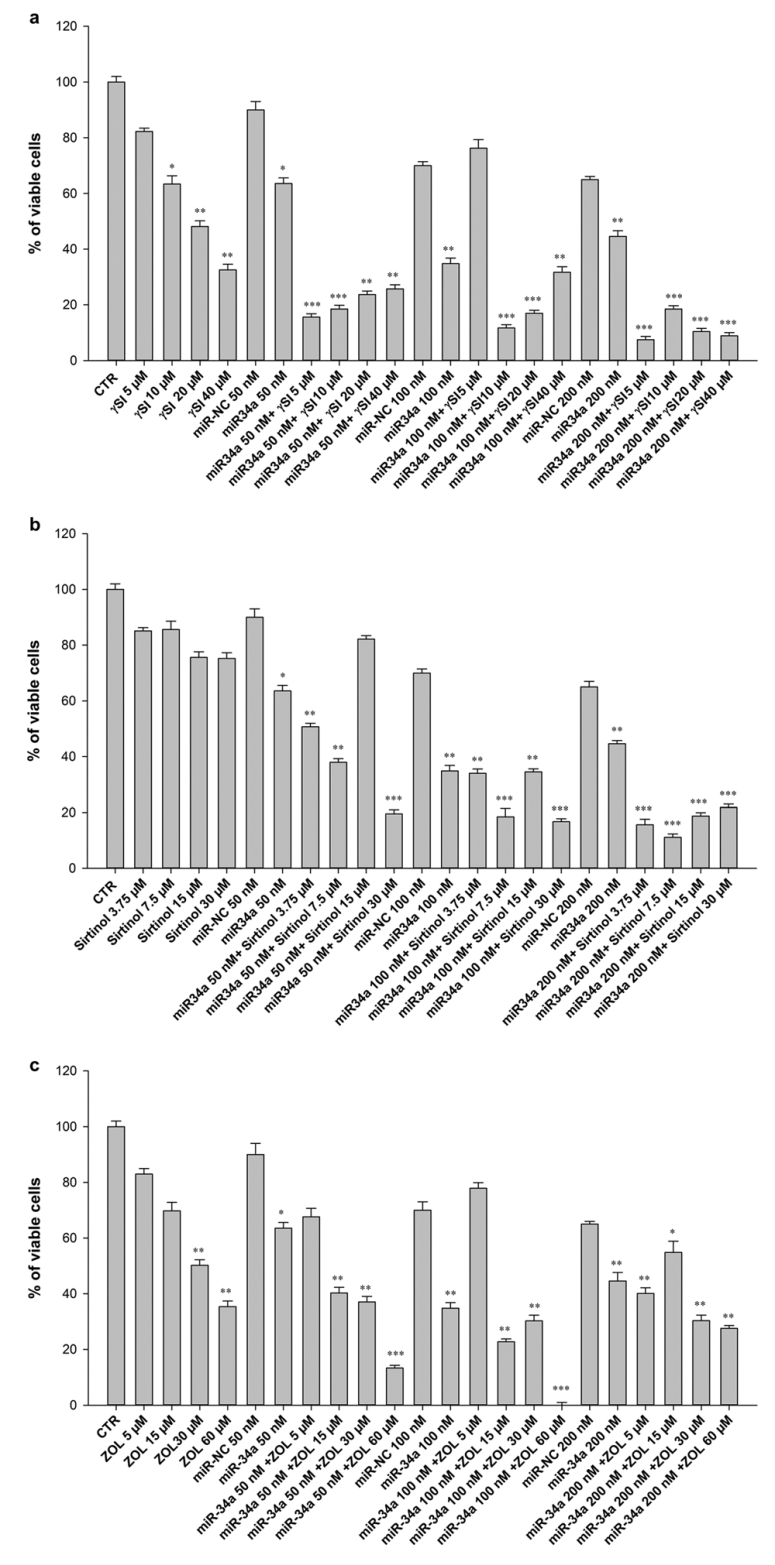
Cellular Viability Assay after miR-34a transfection and anti-cancer drugs treatment. Trypan blue cell assay of MM RPMI 8226 cells transfected with miR-34a or miR-NC and treated with the γSI (**a**), Sirtinol (**b**) and ZOL (**c**) at different concentrations. CTR = untreated cells used as control. Each experiment was repeated at least three times and the data are shown as a mean ± standard deviation (SD). *p ≤ 0.05; **p ≤ 0.01; ***p ≤ 0.001.

Taking into account the observed anti-cancer effect of miR-34a in MM cells^12,17^, we evaluated the effect of this miRNA in combination with three anti-cancer agents. In order to evaluate the transfection efficiency we performed a quantitative RT-PCR after transfection with miR-34a or miR-NC. We observed that cells transfected with miR-34a had an about 60-fold increase of miR-34a expression compared to cells transfected with miR-NC, used as internal control (Supplementary Fig. 1S). Thereafter, we analysed the inhibitory effects on cell viability induced by growing concentrations of γSI XII, Sirtinol or ZOL in RPMI 8226 cells transfected with either miR-34a or miR-NC mimics. As shown in Fig. 1a, survival decrease induced by γSI, after 48 h of treatment, was dose-dependent in RPMI 8226 cells. Moreover, effects greater than 90% of survival inhibition were recorded when cells were transfected with miR-34a and treated with γSI. In details, the most effective miR-34a/γSI combinations were the following: miR-34a 50 nM + γSI 5 μM, miR-34a 100 nM + γSI 10 μM, miR-34a 200 nM + γSI 5 μM; they induced an about 84.4, 88.3 and 92.5% of cell growth inhibition, respectively. Conversely, a clear antagonistic effect was found with the combination miR-34a 100 nM + γSI 5 µM. On the other hand, Sirtinol alone induced only a slight anti-survival effect, while a marked reduction of cell viability was observed with the following combinations: miR-34a 50 nM + Sirtinol 7.5 µM, miR-34a 100 nM + Sirtinol 7.5 µM, miR-34a 200 nM + Sirtinol 7.5 µM and miR-34a 50 nM + Sirtinol 30 µM; they caused an about 62.1, 81.5, 88.9 and 80.5% of cell growth inhibition, respectively (Fig. 1b). As shown in Fig. 1c, treatment with ZOL induced a dose-dependent growth inhibition. Most miR-34a/ZOL combinations showed high inhibitory effects on cell survival, albeit less effective than the other anti-cancer agents.

An about 85% growth inhibition was reached with the combination miR-34a 50 nM + ZOL 60 µM and about 100% with miR-34a 100 nM + ZOL 60 µM. In parallel, both miR-NC transfected and untransfected cells showed similar viability when treated with the anti-cancer agents (data not shown).

Therefore, the analysed combinations exert a powerful cell growth inhibition, enhancing the activity of miR-34a.

### Apoptosis induction in RPMI 8226 cells treated with γSI, Sirtinol or ZOL after miR-34a mimics transfection

**Figure 2:**
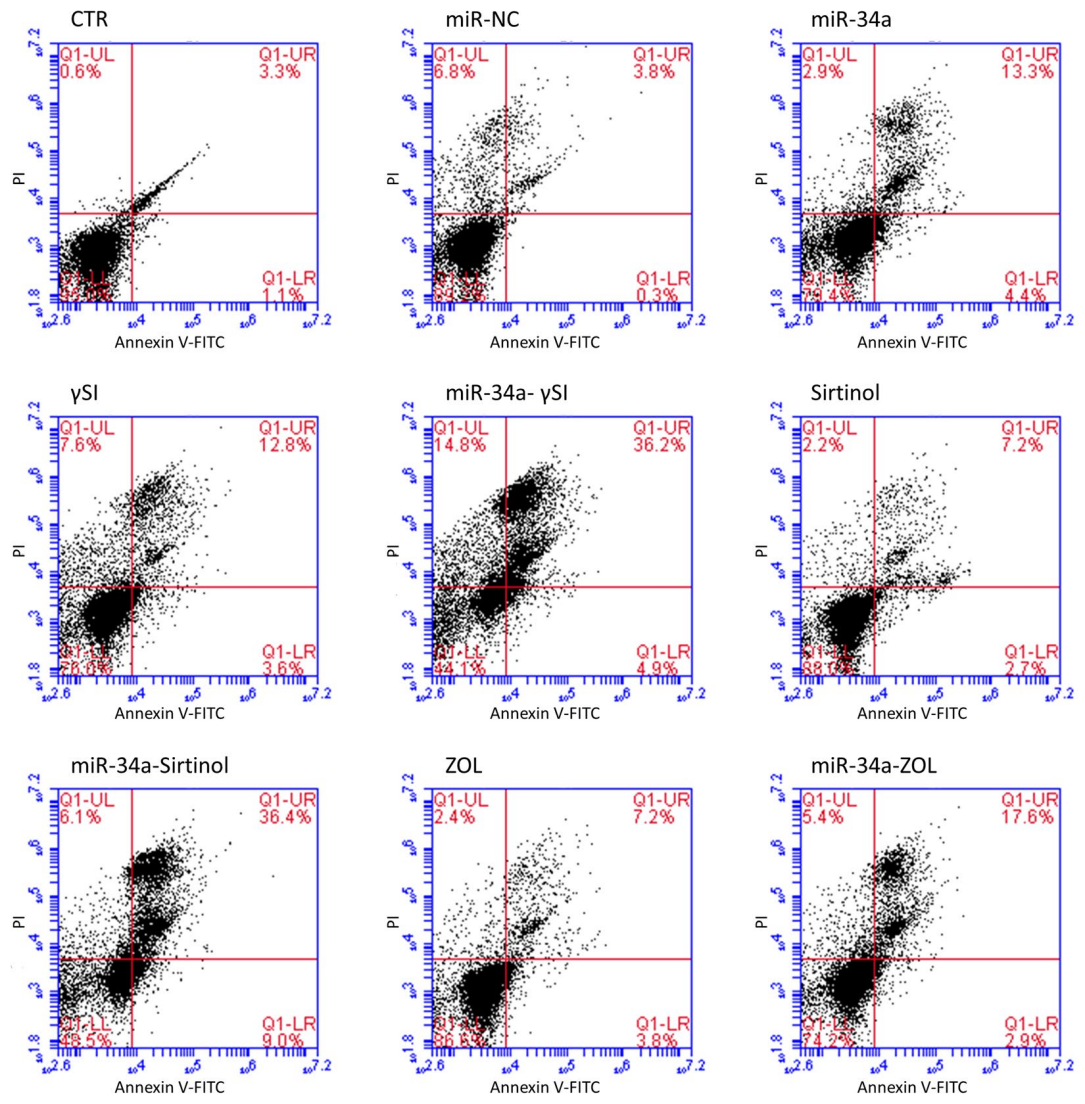
Apoptosis evaluation by FACS analysis. RPMI 8226 cells were analyzed by flow cytometric analysis after transfection with 50 nM miR-34a or miR-NC and treatment, for 48 h, with γSI, Sirtinol and ZOL, as described in “Methods”. UL = Upper Left (necrosis); UR = high to right (in late apoptosis); LL = low left (viable); LR = lower right (early apoptosis). CTR = untreated cells; miR-NC = transfected cells with miR-NC 50 nM; miR-34a = transfected cells; γSI = cells treated with γSI; miR34a-γSI = cells transfected with miR-34a and treated with γSI; Sirtinol = cells treated with Sirtinol; miR-34a-Sirtinol = cells transfected with miR-34a and treated with Sirtinol; ZOL = cells treated withZOL; miR-34a-ZOL = cells transfected with miR-34a and treated with ZOL. The data is representative of three different experiments that have always yielded similar results. The results are shown as cells percentage in the respective quadrants.

Based on previous results on cell growth inhibition, we selected the more synergistic combinations between miR-34a and target-based agents. As described in Methods, after transfection with miR-34a and treatment with anti-cancer agents, the RPMI 8226 cells were stained with AnnexinV-FITC with or without propidium iodide. Flow cytometric analysis revealed that miR-34a (50 nM) transfection induced early apoptosis occurrence in a small % of cell population (4.4%) and late apoptosis in about 13.3% of cells (Fig. 2). Treatment with single agents, specifically 5 µM γSI, 7.5 µM Sirtinol and 60 µM ZOL, caused total apoptosis in about 16.4%, 9.9% and 11.0% of cells, respectively. Enforced expression of miR-34a in RPMI 8226 cells increased apoptosis induction in a synergistic manner when combined with either 5 µM γSI or 7.5 µM Sirtinol. In fact, cells transfected with miR-34a and then treated with γSI or Sirtinol showed a significant increase in the percentage of apoptotic cells (about 41.1% and 45.4%, respectively) compared to untreated (about 4.4%) or cells treated with either γSI or Sirtinol alone (about 16.4% and 9.9%, respectively). Otherwise, the apoptosis induction in cells transfected with miR-34a and treated with ZOL was less significant (only about 20.5%) even if it was higher than in untreated (about 4.4%) or ZOL treated cells (about 11.0%) (Fig. 2).

### Perturbation of the major survival/death pathways by anti-cancer drugs after enforced miR-34a expression in MM cells

**Figure 3:**
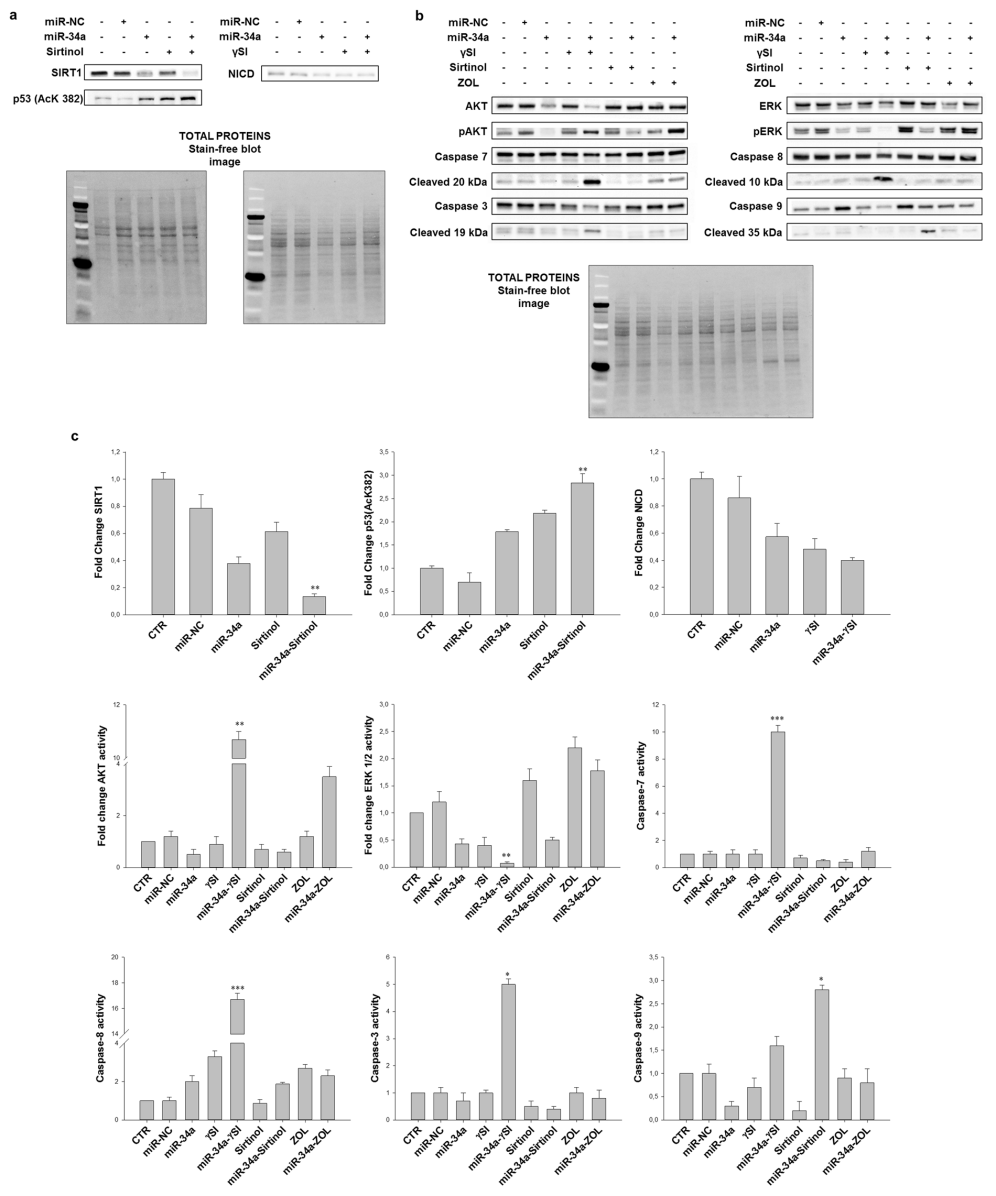
Modulation of the major survival/death pathways by anti-cancer drugs after ectopic expression of miR-34a in MM cells. RPMI 8226 cells were transfected with miR-34a or miR-NC and treated with γSI, Sirtinol and ZOL, as described in “Methods”. After 48 hours treatment, cells were collected for Western blotting analysis. Subsequently, the expression of SIRT1, p53(Ack 382), NICD, AKT, p-AKT, ERK1/2, p-ERK1/2, pro-Caspases 7, 3, 8 and 9, and their cleaved fragments, were evaluated after blotting with specific antibodies, as described in “Methods” (**a/b**). Histograms show the variation of protein levels with respect to total proteins, by Image Lab 5.2.1 ChemiDocTM XRS + (BIORAD). The results are shown as the ratio between the active and non-active forms and compared to control cells levels (**c**). Each experiment was repeated at least three times and data are shown as mean ± standard deviation (SD). *p ≤ 0.05; **p ≤ 0.01; ***p ≤ 0.001.

Based on the attempt to potentiate the inhibition of oncogenic miR-34a targets, we analysed, by Western blotting, the expression of two of them, also main substrates of Sirtinol and γSI: SIRT1 and Notch1intracellular domain (NICD), respectively (Fig. 3a). As expected, SIRT1 inhibition, induced by either miR-34a or Sirtinol, was further enhanced with the combined treatment. This effect was also confirmed by the decreasing of histone deacetylase activity of SIRT1 causes increased p53 acetylation. Also NICD expression, evaluated after miR-34a transfection and/or γSI treatment, was decreased upon each treatment.

The survival-inhibiting and pro-apoptotic effects of miR-34a in combination with the anti-cancer agents, prompted us to study the modulation of two key molecules involved in the regulation of both cell proliferation and survival, MAPK (ERK-1 and ERK-2) and AKT.

We found that miR-34a transfection induced a down-modulation of AKT activity in RPMI 8226 cell line (Fig. 3b,c). Interestingly, γSI treatment following miR-34a replacement increased AKT activity about 10-fold if compared to untreated cells. Moreover, ERK1/2 phosphorylation was significantly reduced in cells transfected with miR-34a or treated with γSI alone (about 60%) and this effect was potentiated (about 90% of decrease) in cells treated with γSI following miR-34a replacement (Fig. 3b,c). Sirtinol treatment did not change significantly AKT activity; however, this drug increased ERK phosphorylation (2-fold if compared to control cells) and this effect was antagonized by miR-34a ectopic expression. Finally, both Akt activity and ERK phosphorylation were increased by ZOL after miR-34a transfection.

On the light of the pro-apoptotic effects induced by miR-34a transfection combined with anti-cancer drugs treatment, we evaluated the proteolytic processing of both initiator (Caspase-8 and -9) and executioner caspases (Caspase-3 and -7). Full length and cleaved caspases come from the same blot as a result of exposures at different times (Supplementary Fig. 2S). Accordingly to apoptosis induction found by cytoflurimetric analysis, we observed that γSI treatment after miR-34a replacement induced a significant increase in cleaved isoforms of both the initiator and executioner caspases (Fig. 3b,c). Even the combination of miR-34a with Sirtinol or ZOL enhanced the expression of the cleaved isoforms of caspases, although to a lesser extent. In details, cell treatment with γSI after miR-34a transfection increased Caspase-8 cleavage of about 17-fold, if compared to control cells, and executioner Caspases-3 and 7 of 5- and 10-fold, respectively. An increase of Caspase-8 activity was also observed in cells treated with ZOL or Sirtinol after miR-34a transfection. However, miR-34a/Sirtinol combination induced a major activation of Caspase-9, about 3-fold with respect to control cells. These data indicate that γSI and Sirtinol treatments enhance the apoptosis induced by miR-34a through the activation of either an extrinsic or an intrinsic pathway, respectively. On the other hands, ZOL induced a detectable activation of the extrinsic apoptotic pathway, but the potentiation induced by miR-34awas less significant compared to those induced by the other two agents.

**Figure 4:**
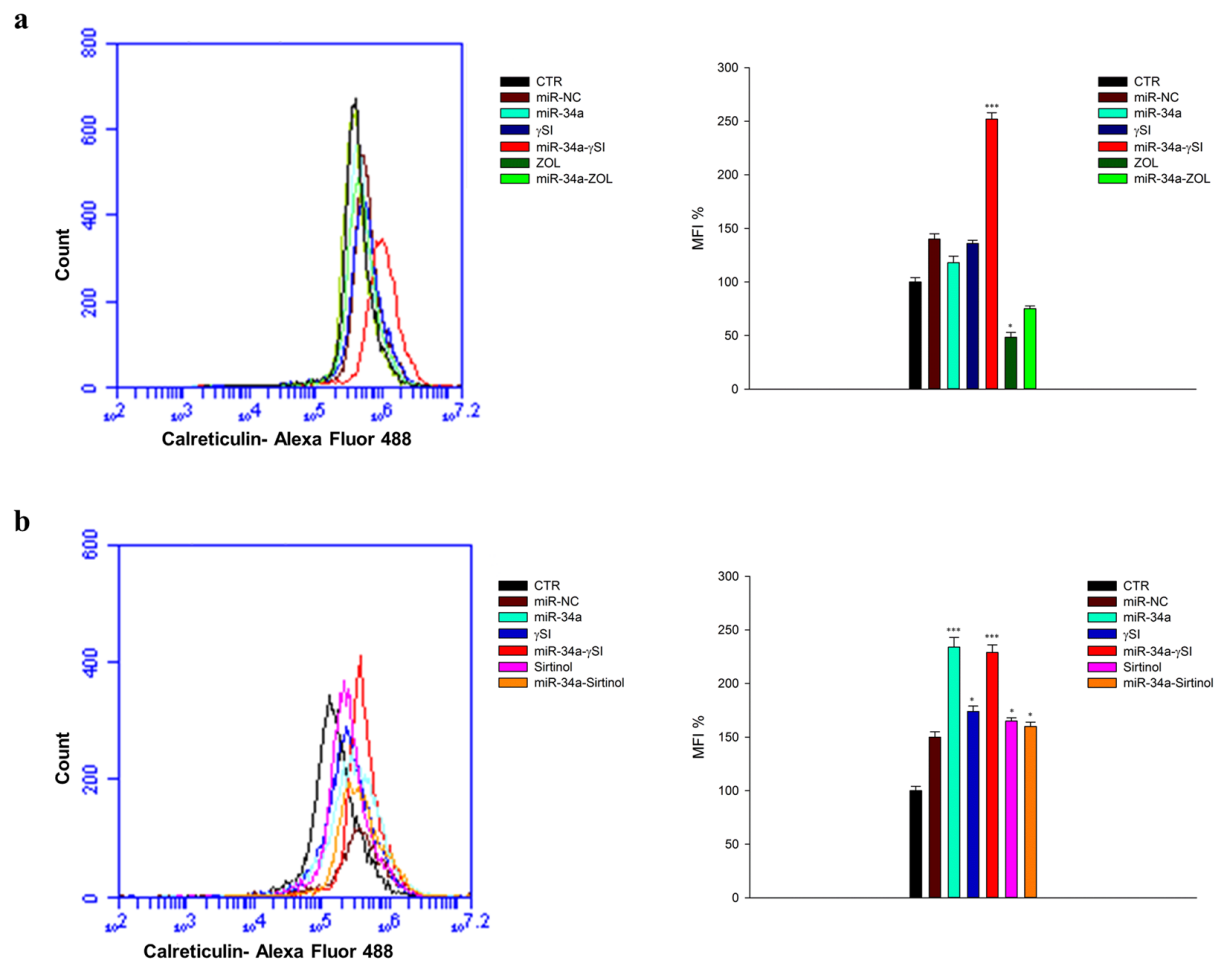
CRT surface expression in RPMI 8226. RPMI 8226 cells were transfected with miR-34a or miR-NC and treated with γSI, Sirtinol and ZOL, as described in “Methods”. After 24 (**a**) and 48 (**b**) hours of treatment the cells were collected and incubated with Calreticulin antibody, as described in “Methods”. Cells were analyzed by flow cytometric analysis and results are shown in histograms, setting control sample equal to 100. The data is representative of three different experiments that have always yielded similar results. *p ≤ 0.05; **p ≤ 0.01; ***p ≤ 0.001.

Caspase-8 activation can induce cleavage of the ER protein BAP31 and conformational activation of Bax and Bak that is paralleled by the secretion of calreticulin (CRT), in turn able to elicit an anti-cancer immune response^32^. On these bases we evaluated CRT cell surface expression in our experimental conditions. We found an about 2.5 fold increase in mean fluorescence intensity (MFI) of CRT expression in miR-34a-transfected cells treated with ɣSI for 24 h, compared to parental cells (Fig. 4a); similar results were also recorded after 48 h (1.7 and 2.4 fold of increase with ɣSI alone or in combination with miR-34a, respectively) (Fig. 4b). Interestingly, miR-34a transfection had no effects on CRT expression at 24 h (Fig. 4a), while it increased CRT expression about 2.4 fold after 48 h (Fig. 4b). Sirtinol did not induce any change of CRT expression at 24 h neither in parental or miR-34a-transfected cells (data not shown), while at 48 h it induced an about 1.5 fold increase of CRT expression in parental cells and no change was observed in miR-34a-transfected cells (Fig. 4b). On the other hand, ZOL decreased CRT expression of about 50% and 25% in parental and miR-34a-transfected cells respectively at 24 h (Fig. 4a), while it did not change CRT expression in parental cells and caused an about 45% CRT reduction in miR-34a-transfected cells at 48 h (data not shown). These data are in agreement with the results about apoptosis induction, confirming the higher pro-apoptotic effect induced by ɣSI and Sirtinol and the strong increase of Caspase-8-dependent pathway induced by ɣSI, that could be paralleled with a possible pro-immunogenic up-regulation of CRT secretion.

**Figure 5:**
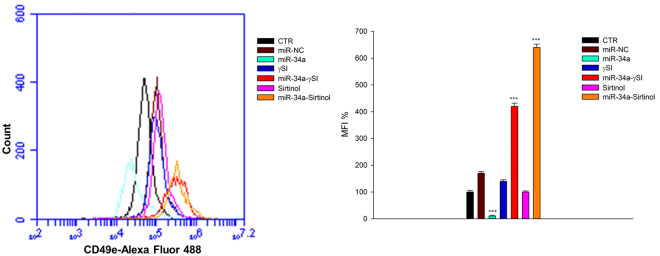
CD49e surface expression in RPMI 8226. RPMI 8226 cells were transfected with miR-34a or miR-NC and treated with γSI, Sirtinol and ZOL, as described in “Methods”. After 48 hours of treatment the cells were collected and incubated with CD49e antibody, as described in “Methods”. Cells were analyzed by flow cytometric analysis and results are shown in histograms, setting control sample equal to 100. The data is representative of three different experiments that have always yielded similar results. *p ≤ 0.05; **p ≤ 0.01; ***p ≤ 0.001.

Interestingly, in the same experimental conditions, we have also observed phenotypic effects induced by both miR-34a and anti-cancer agents on RPMI 8226 cell line. In details, miR-34a transfection alone induced a strong decrease of the maturation antigen CD49e (about 80%) but the treatment of transfected cells with either ɣSI or Sirtinol strongly counteracted this effect causing a potent up-regulation (about 400% and 600%, respectively) of CD49e, if compared to parental cells. Interestingly, the single agents did not induce significant effects compared to untreated parental cells (Fig. 5). On the other hand, ZOL did not induce any effect on CD49e expression (data not shown).

### Effects of miR-34a and ZOL on cell cycle distribution

**Figure 6:**
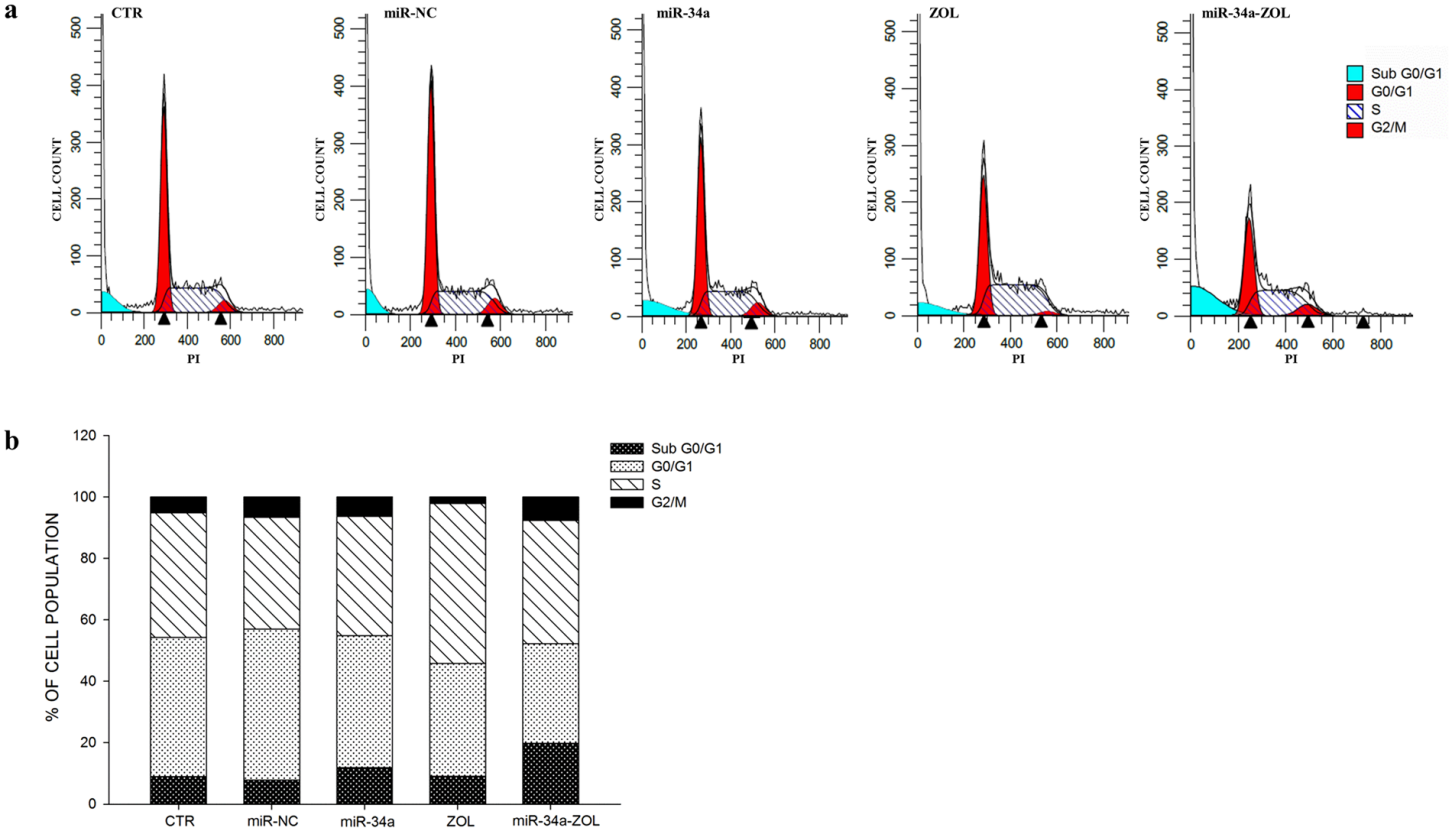
Flow cytometric cell cycle analysis using propidium iodide DNA staining. RPMI 8226 cells were electroporated with miR-34a or miR-NC and treated with ZOL, as described in “Methods”. After staining with PI solution, cells were analyzed on the BD Accuri C6™ flow cytometer. The data were analyzed by the ModFit program from one of three independent experiments (**a**). (**b**) The histogram shows the percentage of cells in each phase of the cell cycle.

In order to evaluate the effects of miR-34a/ZOL combination on RPMI 8226 cell cycle distribution, we performed a FACS analysis after propidium iodide labeling, as described in Methods. Cells treated with ZOL alone showed an increase of S phase of about 1.6-fold compared to control cells. Interestingly, cells transfected with miR-34a and treated with ZOL showed a 2.5 fold increase of “sub-G1” phase compared to either control or cells treated with single agents (Fig. 6). These data were paralleled by the modest increase of the apoptotic occurrence determined by ZOL in transfected cells and require additional investigations in order to better clarify the cell death mechanism induced by ZOL.

### Modulation of autophagic flux in RPMI 8226 cells

**Figure 7:**
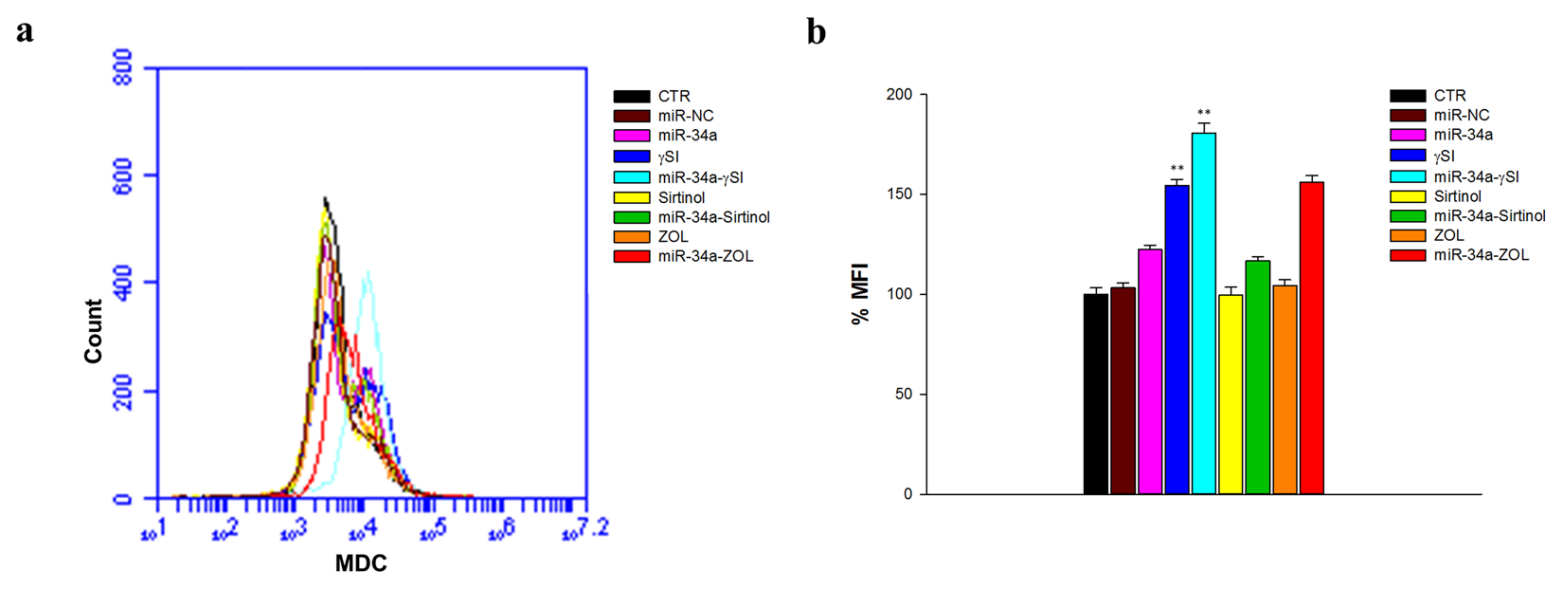
Flow cytometric analysis of autophagy. RPMI 8226 cells were transfected with miR-34a or miR-NC and treated for 48 h with γSI, Sirtinol and ZOL, as described in “Methods”. After MDC staining, cells were analysed on the BD Accuri C6™ flow cytometer (**a**). (**b**) The histogram shows the relative MFI with respect to control sample. The results are shown as means ± standard deviation (SD) for three independent experiments. *p ≤ 0.05; **p ≤ 0.01; ***p ≤ 0.001.

To explain the molecular bases underlying cell death mechanism induced by miR-34a ectopic expression with or without other anti-cancer drugs, we studied also the autophagic flux in RPMI 8226 cell line by flow cytometry after monodansylcadaverine (MDC) staining. The results showed that miR-34a induced only a slight increase in MFI (about 23%) after 48 h from the beginning of the treatment (Fig. 7). Interestingly, ZOL/miR-34a combination induced a significant increase in MFI: about 56%, compared to untreated or single agent treated cells. The treatment with γSI alone also induced a significant increase of autophagic flux (about 54% compared to control cells), which was potentiated by miR-34a transfection. On the other hand, only a slight increase in MFI was observed when cells were treated whit Sirtinol after miR-34a replacement (19%), compared to miR-34a alone (23%). Therefore, ZOL was the most active agent in potentiating the pro-autophagic effects induced by miR-34a.

## Discussion and Conclusion

In the last decade, the outcome of patients with MM has markedly improved due to the introduction of novel agents such as proteasome inhibitors (bortezomib) and immunomodulatory drugs (thalidomide, lenalidomide). However, MM commonly acquires drug resistance leading to relapse of disease^2^. Recently, miRNAs emerged to have a key role in MM pathophysiology and the replacement of oncosuppressor miRNAs provides a promising strategy against tumors^5,6,11,33–35^.

In this study, we focus on the molecular mechanisms of miR-34a-mediated tumor suppression in RPMI 8226 MM cell line and its role in modulating responsiveness to anti-cancer drugs, in particular γSI, Sirtinol and ZOL. Results showed that miR-34a exerts a powerful inhibition of cell viability (Fig. 1) by inactivating two key molecules involved in the regulation of both cell proliferation and survival, AKT and ERK1/2, and induces apoptotic cell death by activating extrinsic pathway (Fig. 3).

**Figure 8:**
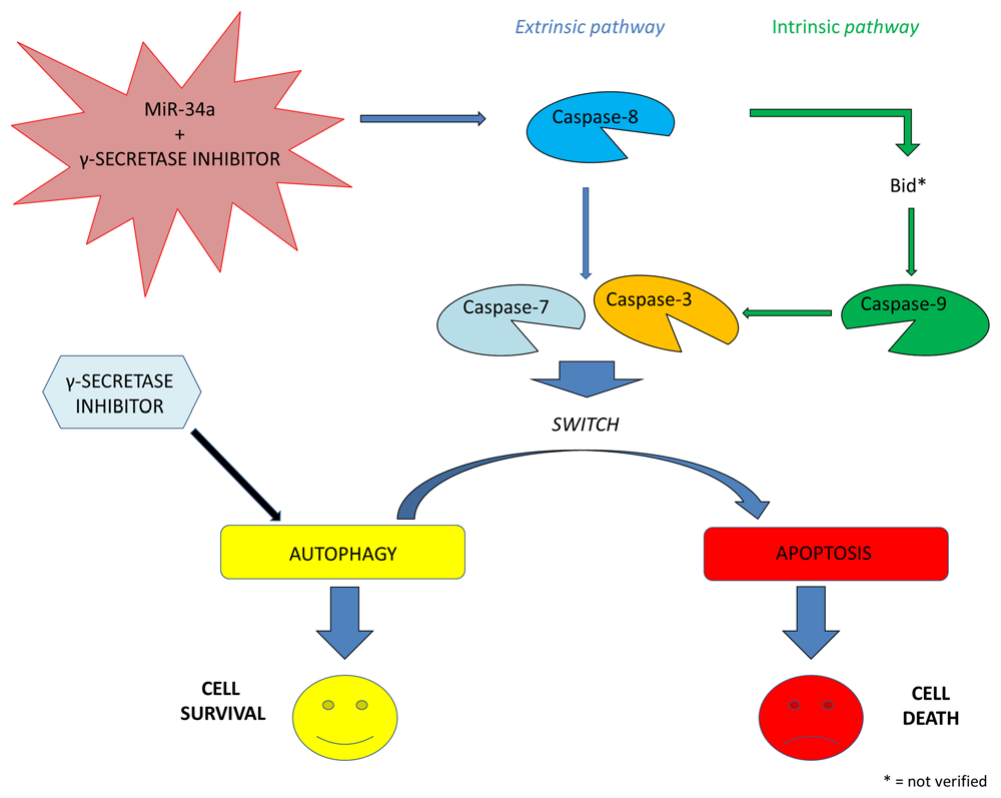
Representative diagram of the possible cell death mechanism. RPMI 8226 cell line treated with γSI showed a significant increase of autophagic flux, but the transfection with miR-34a did not potentiate this effect. On the other hand, enforced expression of miR-34a in cells treated with γSI induced a powerful increase of apoptosis compared to cells treated with the anti-cancer drug alone. We hypothesized that the key player in this antitumor response is the massive Caspase-8 activation that results in the Caspase cascade activation, where downstream effectors Caspase-3 and -7 cleave protein targets involved in cell death. Additionally, Caspase-8 can cleave Bid protein, thus activating the intrinsic pathway of apoptosis which, in turn, enhances the extrinsic pathway. Moreover, Caspase-8 inhibition results in a significant increase of autophagic flux and an increase in cell viability equal to control cells. Our results show miR-34a as a potent weapon to overcome the cytoprotective autophagic mechanism induced by γSI treatment switching cells in an apoptotic status.

Several studies have demonstrated that inhibiting multiple components of the same signaling pathway has greater efficacy compared to targeting individual components by mediating a more complete inhibition of signaling^36^. Moreover, miR-34a has showed to sensitize different cell types to conventional drug treatments^37–40^. Our data demonstrate that miR-34a enforced expression in RPMI 8226 cells significantly enhances the anti-tumor effect of all three agents γSI, Sirtinol and ZOL. All these agents have proven efficacy in inducing growth inhibition in a wide range of experimental models, including MM cells^41–43^. Our data, in agreement with literature, also confirm this trend. Based on the powerful inhibition of cell viability (Fig. 1), we investigated the molecular mechanisms underlying cell growth inhibition in the combination settings. In details, Western blot analysis confirmed Notch1 inhibition by miR-34a and/or γSI treatment (Fig. 3a). Moreover, MM cells treated with γSI after miR-34a replacement showed a significant apoptosis induction (41% if compared to untreated cells) (Fig. 2). Western blotting analysis confirmed apoptosis activation by the proteolytic processing of initiator (Caspase-8 and -9) and executioner caspases (Caspase-3 and -7) (Fig. 3b,c). Based on highly significant activation of Caspase-8 (about 17-fold with respect to untreated cells), we hypothesized that apoptosis was initiated by the death-receptor (extrinsic) pathway leading to caspase cascade activation, where downstream effectors Caspase-3 and -7 cleave protein targets involved in cell death. Additionally, Caspase-8 can cleave the Bcl-2 related protein Bid that translocates into the mitochondria, causing the release of cytochrome c and the formation of the Apaf-1-containing apoptosome. The subsequent Caspase-9 activation stimulates, in turn, the effectors Caspase-3 and -7 and induces cell death^44^. Interestingly, a significant increase of autophagy induction in cells treated with γSI, w/wo miR-34a ectopic expression, was observed after MDC staining (Fig. 7). In the last decade, it has been well established that programmed cell death (PCD) is not confined to apoptosis (type-I PCD) but cells may use different mechanisms of active self-destruction, like autophagy (type-II PCD). Autophagy can leads to cell death through apoptosis by enhancing caspase activation, but it may also act as a cell survival process by acting as a stress response, delaying caspase activation, and removing damaged organelles^45^. In order to understand functional role of autophagy, we evaluated the specific modulation of viability, apoptosis and autophagy in RPMI 8226 cells after enforced miR-34a expression and treatment with γSI upon Caspase-8 inhibition (Supplementary Fig. 3S). Interestingly, the Caspase-8 Inhibitor Z-IETD-FMK drastically reduced the percent of apoptotic cells, considerably increasing the autophagic process. Our data suggest that autophagy activation induced by γSI, further enhanced by miR-34a, occurs as a cytoprotective cell mechanism that switches on apoptosis induction, as summarized in Fig. 8. Additionally, γSI was the most active agent in inducing capase-8-driven apoptosis in miR-34a-transfected cells, if compared to the other two agents. In this light, it was recently proposed that early activation of the endoplasmic reticulum (ER)-sessile kinase PERK leads to phosphorylation of the translation initiation factor eIF2a, followed by partial activation of Caspase-8 (but not Caspase-3), Caspase-8-mediated cleavage of the ER protein BAP31 and conformational activation of Bax and Bak. Finally, a pool of CRT that has transited the Golgi apparatus is secreted by SNARE-dependent exocytosis^32^. MiR-34a transfected cells treated with γSI had a strong increase of CRT expression that was even higher than that one recorded in Sirtinol-treated cells, while ZOL had not significant effects (Fig. 4). These results were paralleled by the finding that γSI was the most effective in inducing Caspase-8 activation, while less or no effects were recorded with Sirtinol and ZOL, respectively. The biomedical implications of the aforementioned findings are the following: γSI potentiation of miR-34a apoptotic effects can be paralleled by potentially useful immunogenic effects on MM cells, such as CRT membrane expression as a signal triggering anti-tumor immunological response.

Regarding Sirtinol, treatment with drug alone induced a weak inhibition of cell viability that was enhanced by miR-34a ectopic expression, also at lower concentrations (Fig. 1). Also the inhibition of Sirtinol target, SIRT, and its deacetylase activity, measured as acetylated p53 levels, were increased by miR-34a/Sirtinol combined treatment (Fig. 3a). Moreover, FACS analysis showed that the combination miR-34a/Sirtinol induces a powerful activation of apoptotic process (about 45.4% of apoptotic cells), if compared to miR-34a or Sirtinol alone (about 17.7% and 9.9%, respectively) (Fig. 2). These data were confirmed by Western blotting analysis. In details, Caspase-9 cleavage suggests the activation of an intrinsic (mitochondria-dependent) apoptotic pathway (Fig. 3b,c). Interestingly, both γSI and Sirtinol had also a potent effect on the expression of the maturation antigen CD49e (Fig. 5) that is a differentiation agent for MM cells and is associated with a better prognosis of MM patients^46^. Notably, miR-34a transfection caused a rebound/protective effect in MM cells with a strong reduction of CD49e expression that was however completely counteracted by both γSI and Sirtinol treatment, with even a final potentiation of antigen expression. Otherwise, no effect on CD49e expression was observed in parental and miR-34a-transfected MM cells treated with ZOL. On this light, we have investigated the effect of the latter agent on the biological and biochemical effects induced by enforced miR-34a ectopic expression. Also in the case of ZOL, a potentiation of the growth inhibitory effects induced by miR-34a was observed in MM cell line (Fig. 1), but this effect was not paralleled by a synergistic effect on apoptosis induction (Fig. 2). On the other hand, no autophagy occurrence was recorded in cells treated with ZOL alone, but enforced expression of miR-34a increased the ZOL-induced potentiation of the autophagic flux (Fig. 7).Cell death mechanisms underlying ZOL plus miR-34a treatments remains to be further investigated. Of note, miR-34a could be implicated in ZOL-mediated actin rearrangement, as described by Koizumi M. *et al.* ^47^. They demonstrated that ZOL induces apoptotic death of MM cells at least in part by interfering with cytoskeletal integrity. To confirm morphological changes induced by ZOL, cells were observed with a phase contrast microscope. Preliminary analysis indicates that after ZOL treatment, RPMI 8226 cells were long and narrow (Supplementary Fig. 4Sc), as compared to control (Supplementary Fig. 4Sa) or miR-34a transfected cells (Supplementary Fig. 4Sb). Enforced miR-34a expression in cells treated with ZOL did not appear to affect cell morphology, but visibly reduced cells number (Supplementary Fig. 4Sd).

In conclusion, our data provide novel information on miR-34a as potential cancer therapeutic in MM, enhancing antitumor activity of anti-cancer drugs, such as γSI, Sirtinol and ZOL. Several studies strongly suggest a potential clinical application of γSI in cancer therapy. However, one of the major obstacles is the occurrence of inhibitors-associated side effects, especially cytotoxicity in the gastrointestinal tract^48^. It is likely that γSIs do not exclusively target the Notch signaling pathways, so, it is plausible that γ-secretase inactivation of may lead to dysfunction of vital organs. Therefore, one of the major challenges for future clinical applications is to balance efficacy and toxicity of γSIs^23^. A way for a prompt clinical translation of γSIs is the reduction of their active dose through combination strategies with other agents with not overlapping toxicity, such as miR-34a. Moreover, we have found that the apoptotic mechanism involved Caspase-8 activation and this effect was paralleled by the activation of potentially immunogenic signals (CRT overexpression) and the increased expression of better prognosis factors (CD49e). Recent studies have also demonstrated that SIRT inhibitors (SIRTis) (i.e. Sirtinol, Cambinol, and EX527) can potentiate the antitumor effect of Histone deacetylase inhibitors (HDACs) in leukemia cells, but not in normal leukocytes and hematopoietic CD34+ progenitors^49^. Moreover, HDACis and SIRTis could also overcome resistance to therapeutic agents in MM^50^. Our study also demonstrated that miR-34a sensitizes MM cells to Sirtinol inducing cell death by mitochondria-dependent apoptosis induction. In the latter case, less powerful effects on both CRT and CD49e were observed, suggesting γSI as the most promising agent to be used in combination with miR-34a. A powerful counteraction of cell survival was found also in presence of ZOL; however, additional investigations to explain the mechanisms involved in this advantageous combination are required.

One of the major challenges of miRNA-based cancer therapy is to achieve specific, efficient and safe systemic *in vivo* delivery. Within oncology, the first miRNA-based therapy approach, MRX34^16^ has entered in clinical testing in 2013. Using a liposome-based formulation, MRX34 is a synthetic double stranded RNA oligonucleotide that can substitute depleted miR-34, thus restoring its oncosuppressive role. Our research group demonstrated that, in experimental model of MM, SNALPs conjugated with transferrin and encapsulating a 2′-O-Methylated miR-34a led to the highest increase of survival in mice, compared with untargeted SNALPs^7^. In the same study, the use of an O-methylated miR-34a, compared to a wild type miRNA, lead to a further increase of the mice survival.

Based on our data and previous studies about miR-34a delivery, the possible co-delivery of miR-34a and γSI in opportunely modified nanocarriers could be of great interest in preclinical models for a prompt clinical translation of the results.

## Methods

### Cell cultures

RPMI 8226 MM cell line, kindly provided by Department of Clinical and Experimental Medicine of the University Magna Graecia of Catanzaro, was grown in RPMI-1640 medium, containing L-glutamine (Gibco, Life Technologies, Carlsbad, CA), supplemented with heat-inactivated 20% FBS (Lonza, Basel, Switzerland), 20 mM HEPES, 100 U/ml penicillin, and 100 mg/ml streptomycin (Gibco, Life Technologies, Carlsbad, CA) and incubated at 37 °C in a 5% CO_2_ atmosphere.

### *In vitro* transfection of MM cell line

Cells were seeded at a density of 112 × 10^3^ cells per cm^2^ and grown in RPMI medium without antibiotics. Electroporation with hsa-miR34a-5p (Ambion, Life Technologies, California, USA) was performed at final concentrations of 50, 100 and 200 nmol/L, using Neon Transfection System (Invitrogen) 1050 V, for 30ms, 1 pulse. An oligonucleotide with a random sequence, miRNA Mimic Negative Control, (Ambion, Life Technologies, California, USA) (miR-NC) at the same concentrations was used as control.

### Real-time quantitative PCR

Cell transfection efficiency was evaluated by Real-time quantitative PCR using ViiA 7 System (Applied Biosystems, California, USA). Cells were transfected with miR-34a or miR-NC and, after 48 h, total RNA from RPMI 8226 cells was obtained by mirVana miRNA Isolation Kits (Ambion, Life Technologies, California, USA) according to manufacturer’s instructions. The integrity, quality and quantity of RNA were assessed by the NanoDrop ND-1000 Spectrophotometer (Thermo Fisher Scientific, Wilmington, DE, USA). Oligo-dT-primed cDNA was obtained using the High Capacity cDNA Reverse Transcription Kit (Applied Biosystems). The single-tube TaqMan miRNA assays (Ambion, Life Technologies, California, USA) was used to detect and quantify mature miR-34a according to the manufacturer’s instructions by the use of the Real-time PCR ViiA7 (Applied Biosystems, California, USA). MiR-34a expression was normalized on RNU44 (Ambion, Life Technologies, California, USA). Comparative real-time PCR (RT-PCR) was performed in triplicate, including no template controls, and relative expression was calculated using the comparative cross-threshold (Ct) method.

### Cell viability assay

Cell viability was analysed by the Trypan blue 0.2% staining (Lonza, Basel, Switzerland). MM cell line was electroporated, as previously described, with miR-34a or miR-NC. After 24 h cells were treated or not with anti-cancer agents, gamma secretase inhibitor (γSI XII, Merck Millipore, Darmstadt, Germany), Sirtinol (Selleckchem, USA) and Zoledronic acid (Zometa; Novartis Pharmaceuticals, Origgio, Italy) at different concentrations. MiR-34a and miR-NC were transfected to a final concentration of 50 nM, 100 nM and 200 nM. Parental cells or miR-34a and miR-NC transfected cells were treated with γSI at final concentration of 5, 10, 20 and 40 µM, with Sirtinol at final concentration of 3.75, 7.5, 15 and 30 µM, and ZOL 5, 15, 30 and 60 µM. Viability assay after 48 h of treatment was performed using Cellometer Auto 1000 (Nexcelom, Bioscience, Lawrence, USA) according to manufacturer’s instructions. Each experiment was performed in triplicate and data were expressed as mean ± SD.

### Western blot analysis

RPMI 8226 MM cell line was electroporated w/wo miR-34a or miR-NC 50 nM and treated, after 24 h, with anti-cancer drugs at the concentrations that showed greater effect on cell survival inhibition. In details, γSI 5 µM, Sirtinol 7.5 µM, and ZOL 60 µM. For cell extract preparation, cells were washed twice with ice-cold PBS/BSA and centrifuged for 30 min at 4 °C in 1 ml of lysis buffer (1% Triton, 0.5% sodium deoxycholate, 0.1 M NaCl, 1 mM EDTA, pH 7.5, 10 mM Na_2_HPO_4_, pH 7.4, 10 mM *phenylmethylsulfonyl fluoride* (PMSF), 25 mM benzamidin, 1 mM leupeptin, 0.025 U/ml aprotinin). Lysates were spinned at 13000 g for 10 min and supernatants were collected. Protein concentration was determined by Lowry method and compared with bovine serum albumin standard curve. Equal amounts of cell proteins were separated by SDS-PAGE using TGX Stain-Free™ FastCast™ Acrylamide Solutions (BioRad, California, USA).The proteins were electro-transferred to nitrocellulose by Trans blot turbo (BioRad, California, USA) and reacted with the different MAbs. Notch1 (D6F11), SIRT1, Akt and p-AKT, p44/42 MAPK (Erk1/2), Phospho-p44/42 MAPK (Erk1/2) (Thr202/Tyr204), Casp 7 (Human specific), Casp 8 (D35G2), Casp 9 MAbs were purchased by Cell Signaling (Cell Signaling Technology, Beverly, MA); Acetyl-p53 (Lys382) by Thermo Scientific (Thermo Fisher Scientific, Wilmington, DE, USA) and Casp 3 by Alexis (Lausen, Switzerland). After incubation with secondary antibodies, the signal was detected using Clarity™ Western ECL Blotting Substrate (BioRad, California, USA) with ChemiDoc XRS+ imaging system (BioRad, California, USA). The amount of the target proteins was normalized to the amount of total protein present in each lane using the stain-free detection method on the nitrocellulose membrane^51^. Image Lab™ Software for quantitative analysis of proteins was used. The error bars shown in the histograms represent the standard deviation from the mean of different image acquisitions in at least 3 different experiments.

### Flow cytometric analysis of apoptosis

RPMI 8226 cells were electroporated w/wo miR-34a or miR-NC at final concentration of 50 nmol/L and treated, the day after, with the anti-cancer agents, as previously described. Caspase-8 Inhibitor Z-IETD-FMK (BD Pharmingen, San Diego,CA, USA) 60 μM, added at the same time of miR-34a transfection, was used in order to understand the mechanism of γSI in inhibiting cell viability. Annexin V-FITC (fluorescein isothiocyanate) (BD Pharmingen, San Diego,CA, USA) was used in combination with a vital dye, Propidium Iodide (PI), to discriminate apoptotic from necrotic cells. After the treatment, cells were collected and centrifuged for 5 min at 1500 rpm. Pellet was washed in PBS and incubated with Annexin-V-FITC and PI in a binding buffer (10 mM HEPES, pH 7.4, 150 mM NaCl, 5 mM KCl, 1 mM MgCl_2_, 2.5 mM CaCl_2_) for 30 min at room temperature, washed and re-suspended in PBS as described by the manufacturer. Analysis of apoptotic cells was performed using BD Accuri C6 flow cytometer (Becton Dickinson, BD, Franklin Lakes, NJ, USA). For each sample, 1 × 10^4^ events were acquired. Analysis was carried out by triplicate determination on at least 3 separate experiments.

### Flow cytometric analysis of CRT and CD49e surface expression

In the same experimental condition, cells were analysed to investigate an anticancer immune response mediated by the secretion of CRT and plasma cell maturity evaluating the very late antigen 5 (CD49e) expression. After 48 h from treatment cells were washed twice in PBS, fixed with paraformaldehyde 3% and incubated over night with anti-Calreticulin antibody (Abcam, UK) or ITGA5 antibody (Proteintech, Manchester, UK). The day after cells were washed in PBS and incubated 1 h with secondary antibody Alexa Fluor 488 goat anti-rabbit IgG (H + L) (Thermo Scientific, Wilmington, DE, USA). Finally, protein cell surface expression analysis was performed using BD Accuri C6 flow cytometer (Becton Dickinson, BD, Franklin Lakes, NJ, USA). For each sample were acquired 1 × 10^4^ events. Each experiment was performed in triplicate and data were expressed as mean ± SD.

### Flow cytometric cell cycle analysis using propidium iodide DNA staining

RPMI 8226 cells were electroporated, as previously described, and treated with ZOL 60 μM. After 48 h of treatment, cells were washed in PBS and directly stained in a PI solution (50 μg/ml PI in 0.1% sodium citrate, 0.1% TritonX-100, Ribonuclease I 100 µg/ml, pH 7.4) for 30 minutes at 4 °C in the dark. Finally, cells were washed in PBS and analyzed on the BD Accuri C6 flow cytometer (Becton Dickinson, BD, Franklin Lakes, NJ, USA). The measure of cell fluorescence reveals distribution of cells in three major phases of the cycle (G1, S and G2/M) and makes it possible to detect apoptotic cells (‘sub-G1’ cells) with fractional DNA content. For the evaluation of intracellular DNA content, 1 × 10^4^ events for each sample were analyzed in at least three different experiments and data were expressed as mean ± SD.

### Flow cytometric analysis of autophagy

The autophagy evaluation was performed after MDC (Sigma, Milan, Italy) staining, a selective marker for autophagic vacuoles (AVOS) and especially autolysosomes. The MM cell line was electroporated w/wo miR-34a or miR-NC at final concentrations of 50 nmol/L. The day after cells were treated with anticancer agents as described above. After 48 h of treatment cells were incubated with 50 μM MDC for 10 minutes at 37 °C and subsequently washed in PBS. The autophagic cells analysis was performed by flow cytometry (BD Accuri C6, Becton Dickinson, BD, Franklin Lakes, NJ, USA). For each sample were acquired 1 × 10^4^ events. Each experiment was performed in triplicate and data were expressed as mean ± SD.

### Statistical analysis

Graphs were obtained using SigmaPlot version 11.0 (Systat Software, Inc., San Jose, CA, USA) and significant differences were determined at P ≤ 0.05 according to Student’s t test.

## Electronic supplementary material

Supplementary Materials
