# Supplementary material for: Evidence of novel miR-34a-based therapeutic approaches for multiple myeloma treatment
Source: Sci Rep. 2017 Dec 20;7:17949. doi: 10.1038/s41598-017-18186-0 (PMC5738363; doi:10.1038/s41598-017-18186-0)
Supplement: Supplementary file 1 — Supplementary Materials [file 41598_2017_18186_MOESM1_ESM.pdf]

Evidence of novel miR-34a-based therapeutic approaches for multiple myeloma treatment

Mayra Rachele Zarone[1], Gabriella Misso[1], Anna Grimaldi[1], Margherita Russo[1], Evzen Amler[2], Maria Teresa Di Martino[3], Nicola Amodio[3], Pierosandro Tagliaferri[3], Pierfrancesco Tassone[3,4] & Michele Caraglia[1,4*]

[1] Department of Biochemistry, Biophysics and General Pathology, University of Campania "L. Vanvitelli"; [2] Institute of Biophysics, 2nd Faculty of Medicine , Charles University , Prague , Czech Republic and Laboratory of Tissue Engineering, Institute of Experimental Medicine , Czech Academy of Sciences , Prague , Czech Republic; [3] Department of Experimental and Clinical Medicine, Magna Graecia University, Salvatore Venuta University Campus, Catanzaro, Italy; [4] Sbarro Institute for Cancer Research and Molecular Medicine, Center for Biotechnology, College of Science and Technology, Temple University, Philadelphia, Pennsylvania, USA.

**Correspondence to: Michele Caraglia, MD, PhD, Department of Biochemistry, Biophysics and General Pathology, University of Campania "L. Vanvitelli", Via L. De Crecchio, 7 80138, Naples, Italy. Phone: +390815665871/4 Fax: +390815665863 email: Michele.caraglia@unicampania.it*

Supplementary Information

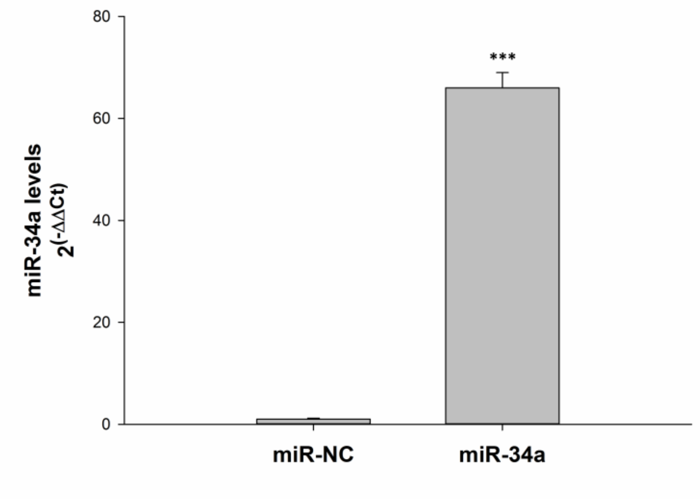

Supplementary Figure 1S. MiR-34a levels after enforced expression. Analysis of miR-34a levels by qRT-PCR in RPMI 8226 cell line after transfection with miRNA Negative Control (miR-NC) or miR-34a, as described in "Methods". Each experiment was repeated at least three times and data are shown as mean ± standard deviation (SD). * $p \leq 0.05$; ** $p \leq 0.01$; *** $p \leq 0.001$.

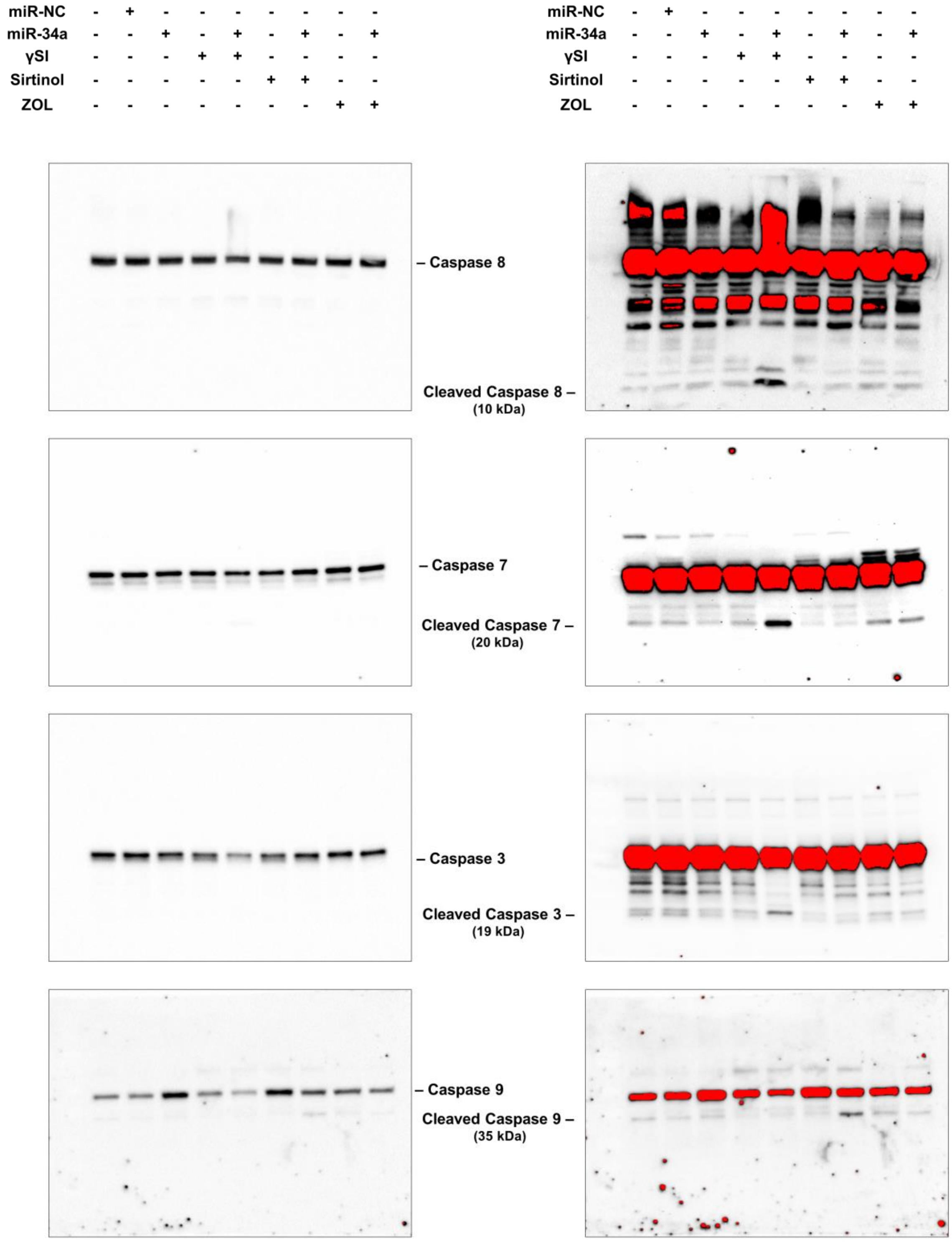

Supplementary Figure 2S. Analysis of caspase activation in RPMI 8226 cells with miR-34a enforced expression after treatment with anti-cancer agents. Western blotting analysis of RPMI 8226 cells transfected with miR-34a or miR-NC and treated with γSI, Sirtinol and ZOL, as described in "Methods". Full length and cleaved caspases come from the same blot as a result of exposures at different times.

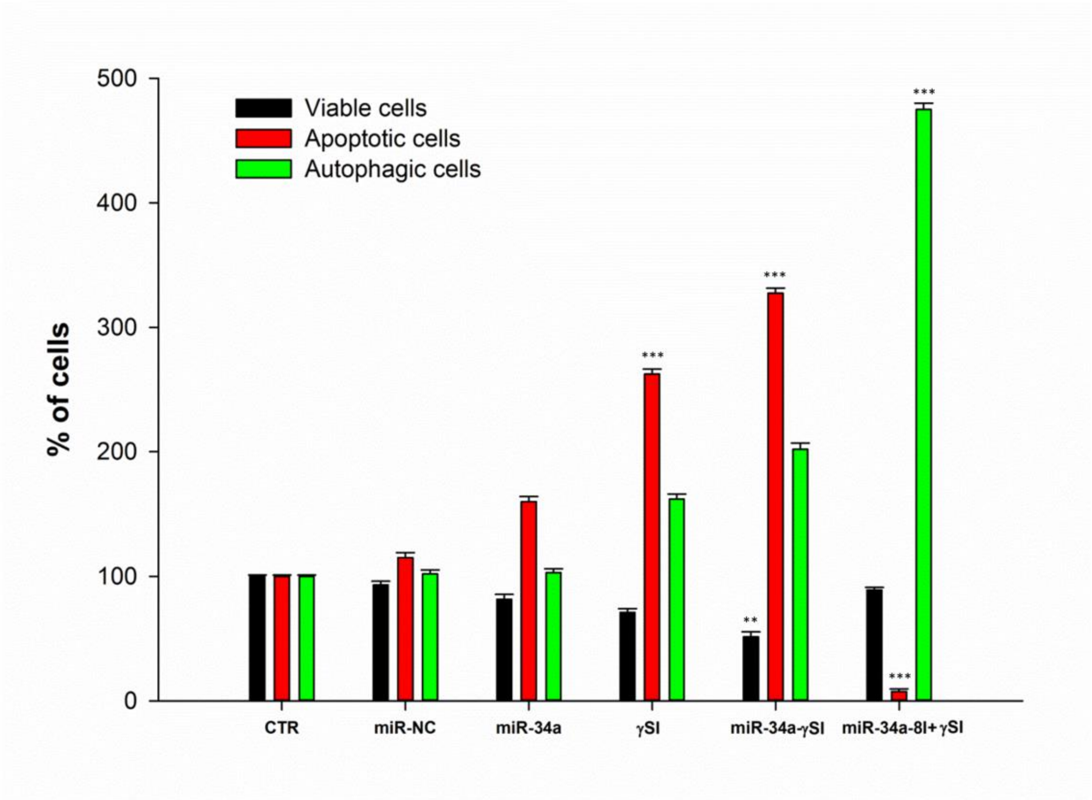

Supplementary Figure 3S. Modulation of apoptosis and autophagy compared to antiproliferative effect mediated by γ-secretase inhibitor in combination with miR-34a. RPMI 8226 cells were transfected with miR-34a or miR-NC and treated with γSI for 48h, as described in "Methods". Caspase-8 Inhibitor Z-IETD-FMK (8I) was added at the same time of miR-34a transfection, as described in "Methods". To evaluate the viability, cells were collected and analysed by trypan blue assay. FACS analyses were performed as described in "Methods". The histogram shows the percentage of viable, apoptotic and autophagic cells compared to untreated cells (CTR) set equal to 100. Each experiment was repeated at least three times and data are shown as mean ± standard deviation (SD).* $p \leq 0.05$; ** $p \leq 0.01$; *** $p \leq 0.001$.

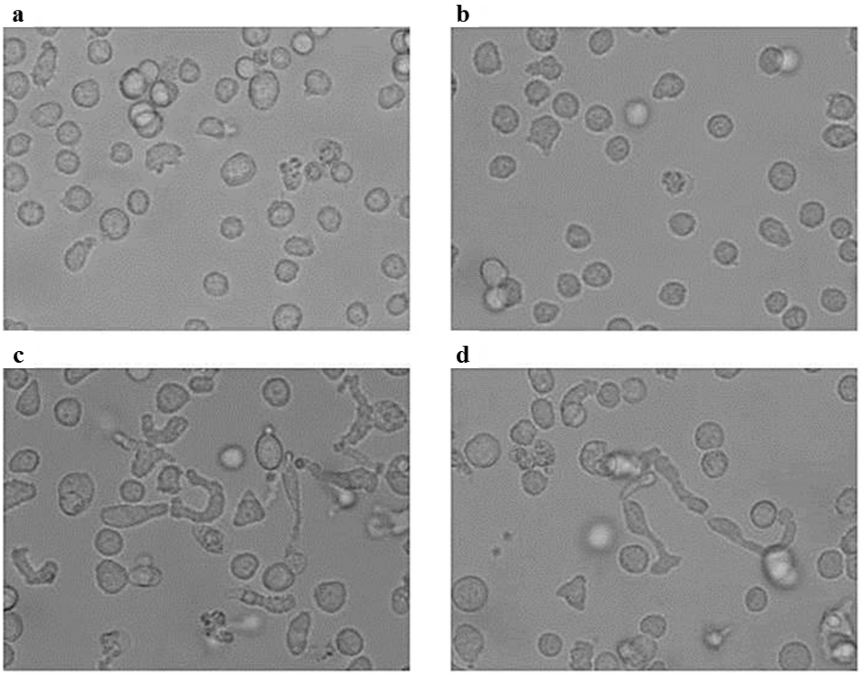

Supplementary Figure 4S. Morphological changes induced by ZOL in RPMI 8226 cell line. RPMI 8226 cells were transfected with miR-34a or miR-NC and the following day treated with ZOL as described in “Methods”. After 48h from treatment, cells were observed with a phase contrast microscope. (**a**) untreated cells; (**b**) cells transfected with miR-34a; (**c**) cells treated with ZOL; (**d**) cells transfected with miR-34a and treated with ZOL.
